# Supplementary figures and images for: Path integration in large-scale space and with novel geometries: Comparing vector addition and encoding-error models
Source: PLoS Comput Biol. 2020 May 7;16(5):e1007489. doi: 10.1371/journal.pcbi.1007489 (PMC7244182; doi:10.1371/journal.pcbi.1007489)

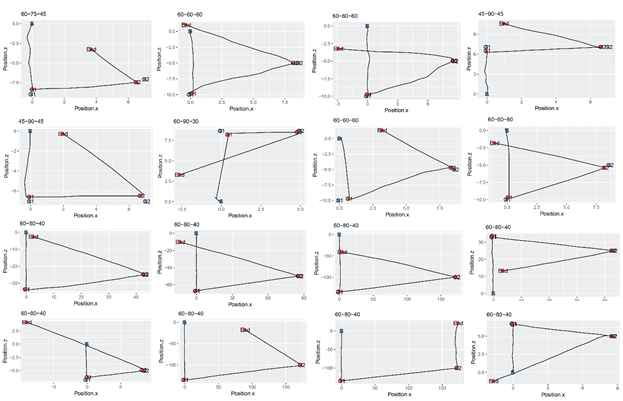

Supplement: S1 Fig — Raw trials from experiment 1 (top 8) and experiment 2 (bottom 8). (TIF) [file pcbi.1007489.s003.tif]

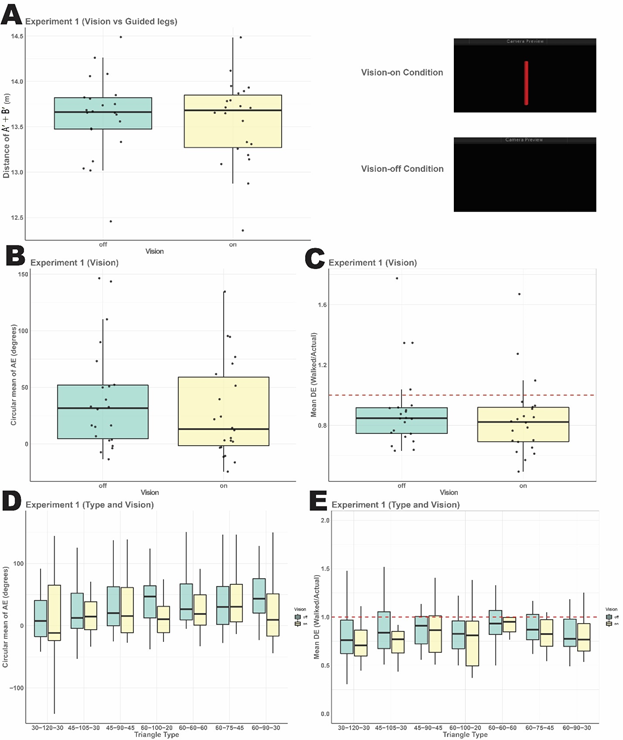

Supplement: S2 Fig — (A) Combined distance walked during guided sides during vision on and vision off trial, showing now differences (t(21) = 1.09, p = 0.288, Cohen's d = 0.336 and BF01>3) (B) Angle error from experiment 1, showing a small but significant difference between vision on and off condition (t(21) = 2.46, p<0.022, Cohen's d = 0.248 and BF10 = 2.54) (C) Distance error from experiment 1, showing a significant difference between vision on and off condition (t(21) = 2.71, p<0.013, Cohen's d = 0.232 and BF10 = 3.94). (D) Angle error from experiment 1, ANOVA significant for triangle type F(6,21) = 2.9, p<0.01, η2 = 0.058 BF10 = 1.72. and Vision F(1, 21) = 4.9, p<0.026, η2 = 0.016 BF10 = 1.16, but not for the interaction between Type and Vision F(6, 21) = 1.454, p = 0.194, η2 = 0.029 BF10 = 0.432. (E) Distance error from experiment 1, ANOVA significant for triangle type F(6, 21) = 5.7, p<0.1.33e-5, η2 = 0.109 BF10>10 and r Vision F(1, 21) = 8.2, p<0.004, η2 = 0.026 BF10>4, but not for the interaction between Type and Vision F(6, 21) = 0.199, p = 0.976, η2 = 0.004 BF10>10. (TIF) [file pcbi.1007489.s004.tif]

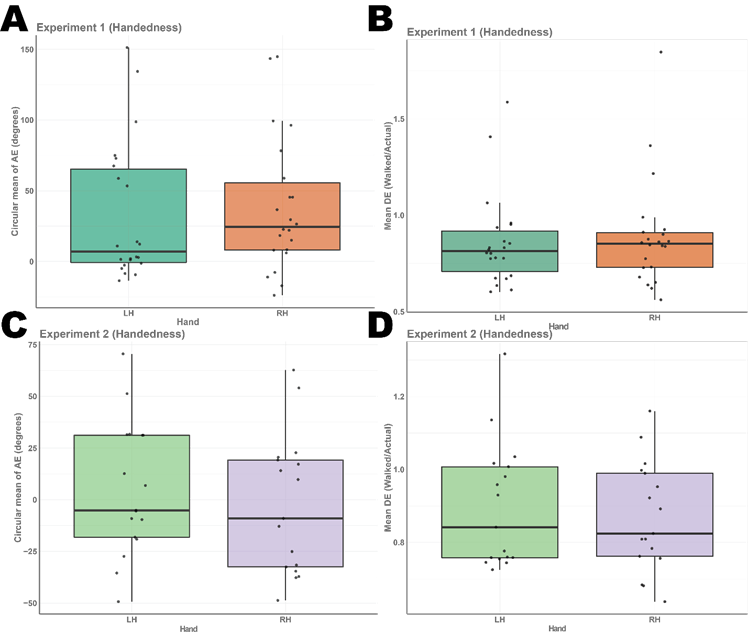

Supplement: S3 Fig — (A) Angle error from experiment 1 which showed no difference between left and right-handed triangles (t(21) = 0.7, p = 0.485, Cohen's d = 0.118 and BF01>3). (B) Distance error from experiment 1, which showed no difference between left and right-handed triangle (t(21) = 1.136, p = 0.268, Cohen's d = 0.103 and BF01 = 2.53). (C) Angle error from experiment 2, again showing no difference between left and right-handed triangle (t(16) = 1.51, p = 0.151, Cohen's d = 0.245 and BF01 = 1.55). (D) Distance error from experiment 2, which showed no difference between left and right-handed triangle (t(16) = 0.724, p = 0.4797, Cohen's d = 0.176 and BF01 = 3.188). (TIF) [file pcbi.1007489.s005.tif]

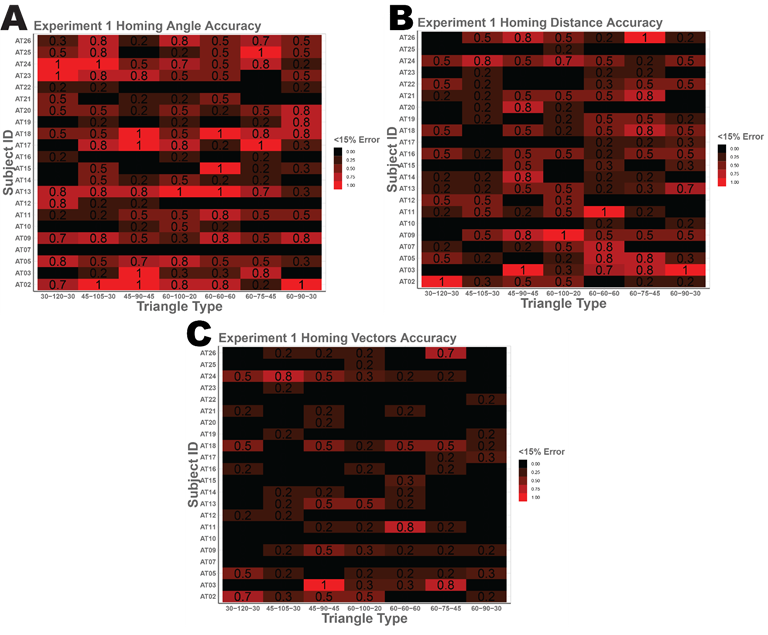

Supplement: S4 Fig — Raster plot (A) showing the percentage of responses with less than 15% angle error (ranging from -27° to 27°) for triangle type (x-axis) and participants (y-axis). Participants were 281.39% more likely to have <15% angle error in their unguided side than <15% total error (angle and distance). (B) percentage of responses with less than 15% distance error (8.5m to 11.5m). Participants are 208.14% more likely to have <15% distance error in their unguided side than <15% total error (angle and distance). (C) percentage of responses with less than 15% angle error (ranging from -27° to 27°) and 10% distance error (8.5m to 11.5m). In (C) we can see that all of participant AT03’s responses for triangle 45-90-45 are less than 15% error for both angle and distance error. And 80% for equilateral triangle (60-60-60) for participant AT11. (TIF) [file pcbi.1007489.s006.tif]

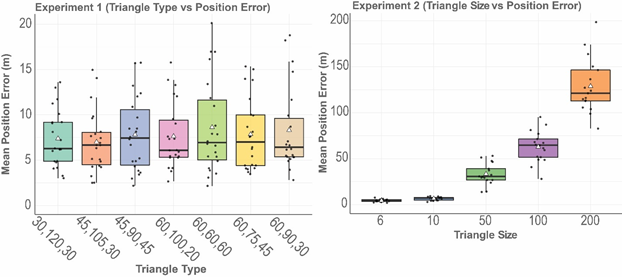

Supplement: S5 Fig — Mean position error (total distance from the participant’s final position and the origin. No main effect of triangle type (left) 1-way ANOVA F(6,21) = 1.34, p<0.24, η2 = 0.06 BF01 = 6.76. (TIF) [file pcbi.1007489.s007.tif]

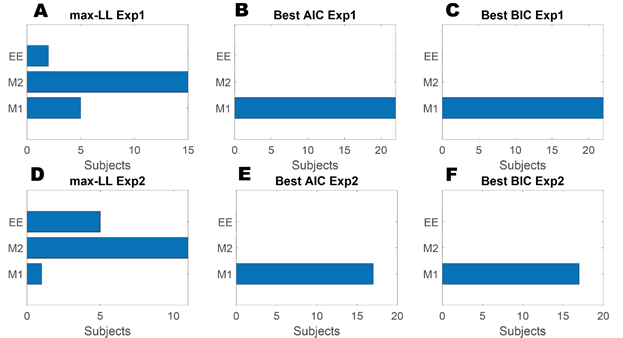

Supplement: S6 Fig — Comparing model fitting of the individual participant’s data. A) Shows best model fit (highest loglikelihood values) for each subject in experiment 1. B&C) Lowest AIC and BIC values across the 3 models for each subject in experiment 2. D) Shows best model fit (highest loglikelihood values) for each subject in experiment 2. E&F) Lowest AIC and BIC values across the 3 models for each subject in experiment 2 (TIF) [file pcbi.1007489.s008.tif]

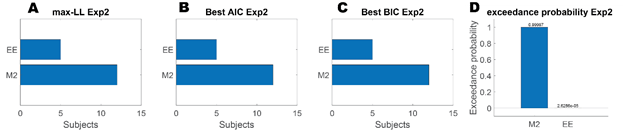

Supplement: S8 Fig — Comparing model fitting of the individual participant’s data. A) Best model fit (highest loglikelihood values) for each participant in experiment 2. B&C) Lowest AIC and BIC values across the 3 models for each participant in experiment 2. D)The exceedance probability of each model for experiment 2. (TIF) [file pcbi.1007489.s010.tif]

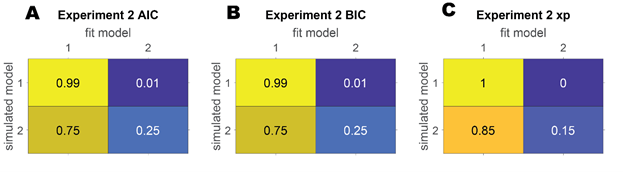

Supplement: S9 Fig — Model recovery confusion matrices. In rows and columns, 1 = Model 1 and 2 = the Encoding-Error Model. Probability ranges from 0 to 1. (A & B) Show best AIC and BIC for Experiment 2 respectively. We see the Encoding-Error does not fit its own simulated data well. (C)The Exceedance Probability for Experiment 2. (TIF) [file pcbi.1007489.s011.tif]

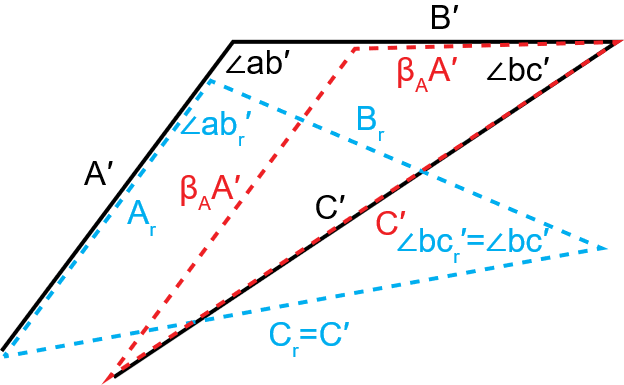

Supplement: S10 Fig — Recreation Fig 1 in Fujita et al. 1993 (modified to employ the names of variable used in our study), as shown with the black solid line which represents the participant’s walked trajectories. The dashed blue lines are the internal encoded representation as predicted by the Encoding-Error Model (these are represented with subscript r). The dashed red line is a rough overlay for the paths predicted by the vector addition model. As shown below, the Encoding-Error model creates sides Ar and Br and angle ∠abr. According to Euclidian properties, this results in side Cr = C and angle ∠bcr = ∠bc. The vector addition model (in red), instead, adjusts A and B accordingly to fit participant’s response C. Thus, the angle ∠ab and ∠bc remain relatively the same in the vector addition model. The critical difference here is that the vector addition models assume a different suboptimal encoding of guided distance for sides A and B. In contrast, the Encoding-Error model assumes the same suboptimal encoding of the guided sides A and B and separate suboptimal encoding of ∠ab such that it preserves Euclid's postulates. *Note that this does not include fitted noise which would slightly change the direction of all three sides. This would in fact make it non-Euclidean such that the total sum of all internal angles does not equal 180. (TIF) [file pcbi.1007489.s012.tif]
