# Supplementary material for: Path integration in large-scale space and with novel geometries: Comparing vector addition and encoding-error models
Source: PLoS Comput Biol. 2020 May 7;16(5):e1007489. doi: 10.1371/journal.pcbi.1007489 (PMC7244182; doi:10.1371/journal.pcbi.1007489)
Supplement: S1 Text — (PDF) [file pcbi.1007489.s013.pdf]

1 S1 Text: Fitting Encoding-Error Model

Encoding-Error Model model has two levels of fit. What we call Level 1 is where we fit the distance and angle function across all trial configurations to find the best value for the slope and y-intercept. These parameters (β_1,β_2,β_3,β_4) would give us the regression to the mean for both angle and distance. In Level 2 we use these parameters to create the encoded values of the guided distance and turn, which are used to create the unguided side using the law of cosine. We fit these values with a new linear distance and angle functions (β_5,β_6,β_7,β_8). We also captured the simulated a noise term similar to Model 1 and model 2

1.1 Level 1

We first need to fit the guided distances (side A and side B) then angles ($\angle ab$: angle between side A and side B) across all triangle configurations.

Klatzky et. al., 1999 page 47 Table 2 shows 2 linear regression fit with 2 parameters used to captures the slope and Y-intercepts of both angle and distance. We use (β_1,β_2) to capture the linear relationship for the guided turn and (β_3,β_4) for the guided side A and side B

Note: β_3 and β_4 are used to fit a linear distance function as the Encoding-Error model assumes a single encoding of distance. Hence Side A and side B are underestimated and overestimated with the same linear function with parameters β_3 and β_4.

$$\angle ab^i = \beta_1 * \theta^i + \beta_4 \tag{1}$$

$$A^i = \beta_3 * |V_1^i| + \beta_4 \tag{2}$$

$$B^i = \beta_3 * |V_2^i| + \beta_4 \tag{3}$$

Where $\angle ab^i$, A^i, and B^i are the triangle configurations for index i (taken from experimental design) and θ^i, $|V_1^i|$, and $|V_2^i|$ are taken from the behavioural data for index i. After we fit angle and distance separately we used the fitted parameters to create new $\angle ab^t$, $\hat{A}^t$, and $\hat{B}^t$ for each subject's trials (denoted by the superscript t).

1.2 Level 2

We use the fitted parameters (β_1,β_2,β_3,β_4) to created the encoded guided angle $\angle \hat{ab}^t$ and side $\hat{A}^t$, and $\hat{B}^t$ for each subject's trial (denoted by the superscript t).

$$\angle \hat{ab}^t = \beta_1 * \theta^t + \beta_4 \tag{4}$$

$$\hat{A}^t = \beta_3 * |V_1^t| + \beta_4 \tag{5}$$

$$\hat{B}^t = \beta_3 * |V_2^t| + \beta_4 \tag{6}$$

Then we used law of cosine to create side C

$$\hat{C}^t = \sqrt{\hat{A^t}^2 + \hat{B^t}^2 - (2\hat{A}^t\hat{B}^t cos(\angle \hat{ab}^t))} \tag{7}$$

When we use law of cosine again to get angle $\hat{\phi}^t$, which is the angle between $\hat{B}^t$ and $\hat{C}^t$

$$\hat{\phi}^t = cos^{-1}(\frac{\hat{C^t}^2 + \hat{B^t}^2 - \hat{A^t}^2}{2\hat{C}^t\hat{B}^t}) \tag{8}$$

We regress the computed angle ($\hat{\phi}^t$) and distance ($\hat{C}^t$) values with linear functions by fitting β_5, β_6, β_7 and β_8.

$$\hat{C^t}' = \beta_5 * \hat{C}^t + \beta_6 \tag{9}$$

$$\hat{\phi^t}' = \beta_7 * \hat{\phi}^t + \beta_8 \tag{10}$$

To then create our $\hat{V3}^t$ components, which is the configural homing vector predicted by Encoding-Error model.

$$\hat{V_{3}}_x^t = \hat{C^t}' cos(\hat{\phi}^{t\prime}) \tag{11}$$

$$\hat{V_{3}}_y^t = \hat{C^t}' sin(\hat{\phi}^{t\prime}) \tag{12}$$

Then we use the maximum likelihood approach similar to Model 1 and Model 2, where ${V_3}_x^t$ is participants data for side D

$$LL_x = -\sum_{t=1}^{T} log(\frac{1}{\sqrt{2\pi\hat{\sigma}^2}} \exp \frac{({V_3}_x^t - \hat{V_{3}}_x^t)^2}{(2\hat{\sigma}^2)}) \tag{13}$$

$$LL_y = -\sum_{t=1}^{T} log(\frac{1}{\sqrt{2\pi\hat{\sigma}^2}} \exp \frac{({V_3}_y^t - \hat{V_{3}}_y^t)^2}{(2\hat{\sigma}^2)}) \tag{14}$$

Where sigma scaled with square root of distance walked:

$$\hat{\sigma} = \beta_9 \sigma(\sqrt{V_1^2 + V_2^2}) \tag{15}$$
