# Supplementary material for: Path integration in large-scale space and with novel geometries: Comparing vector addition and encoding-error models
Source: PLoS Comput Biol. 2020 May 7;16(5):e1007489. doi: 10.1371/journal.pcbi.1007489 (PMC7244182; doi:10.1371/journal.pcbi.1007489)
Supplement: Attachment — Submitted filename: Harootonian et al., PLOS Comp Biol Review 2019.pdf [file pcbi.1007489.s015.pdf]

Comments to the authors:

The manuscript represents a significant technical advance paired with very nice behavioral data and modeling that improves our understanding of path integration in humans over long distances. The manuscript sets up vector addition models, already used in animal locomotion, as a simpler and yet more flexible and maybe more accurate alternative to the encoding error model. Data from these large-scale path integration trials allow the subtleties of these models to be more concretely examined. While I like the manuscript and would recommend it to be published in PLOS Comp Biol, I have several comments that I would like addressed.

Major comments:

1. I was possibly having a particularly dense moment, but I was at page 11 of the paper when I finally realized that the terms ‘guided leg’ and ‘unguided leg’ did not mean the participant’s legs but the epochs of the experiment. I very much dislike the use of the term ‘leg’, especially in a walking experiment. Instead, I would suggest using epoch, phase, portion, side (of triangle) or some other similar term.
2. In the abstract and at a couple of places in the manuscript, you mention that the distances were kept ‘relatively constant’ in experiment 1, and angles were kept ‘relatively constant’ in experiment2. Why were these only relatively constant and not designed to be exactly alike? Are you referring to the participants’ actual trajectory in the guided portion, which slightly differs from the ideal trajectory? If so, that was not clear to me as I read the paper.
3. Similarly, lines 338-339. “...maintained a scalene triangle shape, thus leaving angle as comparatively constant as possible”. Why comparatively? It should be possible to literally scale the same triangle to get whatever distance required, maintaining exactly the same angle.
4. There is some conflation between the terms ‘homing’ and ‘path integration’ in the paper. Homing is the behavior, and path integration is the computation that could potentially result in homing. It would be worth mentioning this difference in the manuscript.
5. Line 100-106. Here you introduce the encoding error model for the first time, and state the assumptions of the models, but yet do not state what the model actually does. I think a brief description of the model is warranted here, perhaps similar to what you later state in the Discussion, lines 566-568.
6. Lines 251-264: Why does the triangle type need to have an effect on both angle **and** distance errors for it to be deemed significant? You do mention elsewhere that it is possible that some sensory modalities have more influence on linear vs. angular motion estimates. Indeed, in these experiments, distances are more

likely underestimated and angles are more likely overestimated. I am unsure why the low effect size on angle errors discounts the result of the effect of triangle type on distance errors. Also, extending the same logic, wouldn't size of the triangle be insignificant since it did not have an effect on both angle and distance errors?

7. Page 14: I think that the models need to be more clearly enumerated. Model 2 is mentioned (Line 294) before Model 1 is, even though model 1 is what you are talking about in the previous paragraph.
   Suggestion: "We employed two versions of a simple vector addition model to fit our results. Model 1 assigns weights to legs A and B, and predicts the distance and direction of leg C. (Equation 2). Model 2, in addition, includes a weighted influence of the participants' past trial history (Equation 5). We compared these two vector addition models to the encoding error model (Fujita et al., 1993)."
8. You use the variables **α** and **β** both to refer to parameters in the vector addition models and also angles between the legs in the task. I would suggest using different variables to maintain consistency and clarity.
9. One key way in which your experimental setup differs from real-world walking is the lack of linear vestibular cues. This is an unavoidable trade-off of being on an omnidirectional treadmill, but I feel like this needs to be explicitly stated and discussed somewhere in Pages 24-25. For example, you could state that desktop VR preserves optic flow, but lacks other path integrative cues. VR with a treadmill would have proprioceptive and motor efference copy signals, but not vestibular cues. VR with omnidirectional treadmill will still lack linear vestibular cues, but preserve angular vestibular cues.
10. In lines 573-574, you mention that the parameters of the linear functions of the encoding error model may not generalize across participants. However, why would the linear relationship between the guided leg and the encoded vector in the vector addition model generalize across participants? In general, I don't think parameter generalization across participants is necessary for a good model, the model structure being preserved and having predictive power across participants is more important.
11. Line 682: "Combining the first two suboptimalities…" which are the two? Only one suboptimality (over or under-weighting) is mentioned before this. I assume you mean noise as the other, but that is not mentioned.
12. I am confused by Equation 3. Why is the noise centered around x_C^t? There is almost always a consistent bias of underestimating distance, and so sampling around the optimal vector x_C^t should not be correct. Do you intend to say that you sampled around the mean vector for each participant?

13. χ and x are very similar-looking symbols and make Eqns. 5 and 6 much less clear. Please use another variable instead of χ to denote linear combination of past vectors.
14. What is $\boldsymbol{\beta}$ in Eqn. 7? I assume it is [$\boldsymbol{\beta}_A$, $\boldsymbol{\beta}_B$, $\boldsymbol{\beta}_\chi$], but please list that explicitly.
15. I would suggest using something other than the white triangles to represent mean in Figs. 2, 4, 5, 6. The marker is low-contrast and not easily visible.
16. Why are the angle errors so tightly clustered in the simulated model data? Figs. 4D, 5D, 6D
17. Figures need more formatting and consistency in font sizes. E.g.
    a. Subfigure names A-H are too large in Figure 1
    b. Fig 1. C,D are clearly horizontally squished.
    c. Labels G1, G2 etc. need to be separated from the markers in Fig. 1 G, H
    d. Axes labels and legends need to be larger and consistently sized, e.g. in Figs. 1G, 1H, 2, 4, 5, 6
    e. Units of quantities in axes labels, e.g. Figs. 1G, H

Minor comments:

1. Line 73: Path integration in cats does not have a citation.
2. Line 76-77: “research on path integration has often been neglected in favor of situations in which visual input provides sufficient information to solve most navigational tasks, such as in desktop virtual reality.” I think it should be mentioned here that vision is not completely lacking in path integrative cues. Optic flow provides a strong velocity cue which can be used for path integration. However, in the real world, the effects of visual landmarks and optic flow cannot be easily decoupled. Virtual reality is one of the ways of accomplishing this.
3. Line 90:I would suggest using the term linear algebra instead of matrix algebra. The properties that you mention are linear transformations, matrices are simply a way of representing them and manipulating them.
4. Line 97: “and then must return” -> “and then the participant must return”.
5. Line 111: “...paths that crossed each other included in Klatzky et al. 1990” -> “...paths that crossed each other.”
6. Line 150: pathway -> pathways
7. Line 156: What do you mean by "single value encoding” here? The terminology is used but never explained.
8. Line 165: infinite large spaces -> infinitely large spaces.
9. Line 167: over shoot -> overshoot
10. Line 185: set-up -> setup
11. Line 209: participants -> participants’

12. Line 212: 0.87 +/- 0.5, which was, on average, close to the the correct response of 1. This is a very qualitative statement. What does the term ‘on average’ mean here? It is already stated that 0.87 is the average value.
13. Line 236: little effect the direction -> little effect on the direction
14. Lines 324-326: The sentence structure is confusing. Please clarify.
15. Line 423-424: 5 subject data -> 5 subject’s data.
16. Line 638: controller -> controllers’
17. Hand-held vs. handheld. I am not sure which one is more accurate, but please stick to one.
18. Line 698: x_C^t should be x_D^t
19. Line 759: templets -> templates
20. Line 791: the15m -> the 15 m
21. Consistency in citations. In various places you have “Author et al., 2000”, “Author et al. 2000”, “Author1 & Author2 2000”, “Author1, Author2, Author 3, & Author 4, 2000” etc. Please check the style guide for PLOS and format citations according to that.
22. Fig. 1 caption: The blue denotes -> the blue point denotes
23. Fig 1F caption: templets -> templates, experiment 1 -> experiment 2
