# Supplementary material for: Path integration in large-scale space and with novel geometries: Comparing vector addition and encoding-error models
Source: PLoS Comput Biol. 2020 May 7;16(5):e1007489. doi: 10.1371/journal.pcbi.1007489 (PMC7244182; doi:10.1371/journal.pcbi.1007489)
Supplement: Attachment — Submitted filename: ResponsetoReviewers.pdf [file pcbi.1007489.s016.pdf]

Reviewer 1

Major

R1.1) “More clarity is needed in the introduction to make the distinction between the vector addition and Encoding-Error Models....In addition, in the introduction it seems like the authors are confounding homing vector models and vector addition models, which are somewhat different. A homing vector (also called a continuous strategy), as it has been defined in the literature (e.g. Fujita et al, 1990; Wiener et al., 2010), is a continuous updating of the animal’s location relative to home, with a single vector representing the relationship to home. This model is history-free in that it does not (in theory) track anything about the path itself, just the return vector. Thus, a vector addition model is not quite the same thing as a homing vector model, because it requires memory of the vectors traveled during triangle completion. In contrast, the Encoding-Error Model – and, from the description, the vector addition model – is a configural model, in which the shape of the outbound path is remembered. Most of the models described in the introduction are also configural models, not homing vector models. So while I think it is completely a good and valid question to compare vector addition and Encoding-Error Models, the distinction of these two within the family of configural models needs to be made clear, as well as the distinction with the family of homing vector models. This issue pops up again in the discussion on page 26 – again, more clarity on the distinction would help.”

We apologize for the insufficient explanation which led to some confusion about the two different models. We have attempted to improve on our explanation of the Encoding-Error Model and Vector Addition Model as well as contextualizing both models within the larger family of configural models. In addition, we moved the section that contrasts the two models to the final paragraph of the introduction after we describe the model based on vector addition. We hope that these additions will help clarify differences between the two models. Accordingly, we have added the following sections to the Introduction:

Encoding-Error Model
“…Configural Models, in contrast, suggest a continuous encoding of the outbound paths (guided path) which are then manipulated using trigonometric or vector calculations to compute a return trajectory (configural homing vector) when required. One of these models, the Encoding-Error Model, suggests that systematic errors in the return path are due to errors in encoding of the outbound paths and their relative configuration [31][32]… This configural model of path integration uses the law of cosines to compute the third side of the triangle given the distance and angles encoded during first two sides (which are the guided portions). Therefore, it is assumed that all systematic errors are accumulate during encoding...*”*

Vector Addition Model
"To attempt to capture the pattern of errors and history effects in the triangle completion task in human participants, we created a simple yet novel configural model based on vector addition often used to understand path integration in other species [24][23]. The model assumes the outbound path is encoded as discrete sets of vectors, such that individual vectors can be used to compute new trajectories such using as a configural homing vector. This model allows for a smaller set of required assumptions and free parameters when compared to Encoding-Error Model as well as a physiologically plausible mechanisms by means of head direction accumulation [26]..."

In addition, we have now tried to be clearer about the theoretical differences between Encoding-Error and vector addition models. We now clarify and setup the theoretical differences between continuous models and configural models in significantly more detail. To avoid confusion, we now refer to the homing vector created in continuous models as a "continuous homing vector" and homing vector created in configural models as "configural homing vector."

"Past experiments on path integration have often involved a path completion task in which the navigator is guided in physical space and must return using the shortest trajectory back to the origin with no guidance [2, 21,22]. Behavioral results both in vertebrates and invertebrates show a systematic bias in the return trajectory which is independent of random accumulated noise [23-27]. Two sets of computational models have been proposed as potential strategies participants might use, in some form, for path integration. The Homing Vector Model (also called a continuous strategy) assumes that the navigator does not encode information about the outbound path but rather continuously updates their current position relative to the origin by computing a continuous homing vector. To capture systematic errors in return paths, Homing Vector Models assume different variations of memory decay or leaky integration which are in theory independent of outbound path configurations [28-30].

Configural Models, in contrast, suggest a continuous encoding of the outbound paths (guided path) which are then manipulated using trigonometric or vector calculations to compute a return trajectory (configural homing vector) when required. One of these models, the Encoding-Error Model, suggests that systematic errors in the return path are due to errors in encoding of the outbound paths and their relative configuration [31, 32]. Recent work, however, suggests that execution errors (during the unguided path) make the largest contribution to systematic errors [33]. Furthermore, Wiener et. al. systematically compared configural and continuous updating strategies by explicitly telling participants to focus on the outbound path or the origin. The results suggest that participants can deploy either configural or continual strategies during a path completion task [34]. Given the widespread application of configural models to both human and non-human path integration [23, 24, 26, 31, 32, 35] and the evidence that humans employ both configural and homing strategies [34], we directly compared

three different configural models of path integration to better understand the patterns of errors that accumulate as participants dead-reckon.”

R1.2) “The results of Exp 1 are pretty clear and the design is easy to follow, but I disagree with the interpretation. The authors found significant differences by triangle type in the angular error and distance error. The conclusion that shape does not contribute to errors just doesn’t follow from those results. It does seem like shape might not contribute to systematic errors – or at least, not systematic errors that the authors can determine. There likely are systematic errors but they are just not obvious. But shape clearly is important and makes a difference to the errors in triangle completion. The authors seem to acknowledge this themselves in line 347, when saying that the consistency of the configuration was important for Exp 2.”

We thank the reviewer for bringing up this point. After careful consideration, we agree that our interpretation was too strong regarding the effect of triangle shape on path integration. We have therefore changed the Results section to be more objective and focused on the statistics. Additionally, we have changed the discussion section to reflect on the statistics and offer three plausible explanations for our findings.

Results section for Experiment 1
".... While we did not find a consistent effect of triangle type across angle and distance errors, it is clear the triangle type contributed significantly to the patterns of participant errors. For example, the isosceles triangle (30,120,30) showed the lowest mean angle error (10.96°±9.11°) yet the equilateral triangle demonstrated the lowest mean distance error (0.985±0.062). Whether participants were relying on triangle templates, abstract geometric cues, or instead repeating identical/similar past paths in working memory, is an issue we return to in the Discussion… Together, these findings suggest that while geometric features (angle and distance) contribute to participant errors, it is not clear how these features are combined (or if at all) to create and deploy triangle templates."

Discussion section Experiment 1
" In Experiment 1, we found that the triangle type tested had an influence on participants’ distance and angle errors on the unguided side. We found that the equilateral triangles showed significantly lower distance error and similarly, we found a weak tendency for the isosceles triangle angles (30,120,30) to show lower angle overestimation. All triangle types observed in Experiment 1, however, showed an angle overestimation and distance underestimation. The question of whether the differences in distance and angle errors observed are due to participants using triangle templates is one we sought to answer in this study.

We believe that If participants were using a triangle template, then we would be able to observe accurate distance and angle estimations for one triangle type. For example, it might be possible to predict that equilateral triangles or right triangles would

be overall more accurate than scalene triangles. This is because these geometries are far more regular and potentially easier to encode holistically, particularly given their influence on visually-guided navigation [53]. While we did not find one triangle type to have lower distance and angle errors at the group level, there may be several explanations for how triangle type had an influence on participants' performance.

First, we found two participants who showed a high degree of accuracy for both distance and angle on equilateral and right isosceles triangles (Supplementary Figure 4C). This suggests that some participants may have used a strategy that involved deploying templates. Unlike Wiener et al. 2011 [34], we did not explicitly ask participants to focus on the outbound path or the origin, which in turn could possibly have affected some participants' likelihood of spontaneous using a template. Our results, in this way, build on other studies showing individual differences in human navigation studies [54-56].

A second explanation for how triangle type may have influenced participants' performance focuses on the possibility of using abstract geometric features. Studies in young children suggest that rather than using room geometry to navigate, children use certain geometric features (i.e. the distance between walls) to reorient themselves [57, 58]. In our task, participants were not directly exposed to the geometric regularities of the path but rather they had to "trace" it by walking it. This process may conserve some geometric features and not others, which may in turn influence the pattern of errors. In particular, because participants could not directly perceive the entire shape, they may have attempted to guess at it, and if their memory for a side was inaccurate, this would affect their memory for the entire shape. Thus, while we could not determine any correlation between patterns of errors and any obvious geometric features (triangles with an acute or obtuse and right angle), there may be other features that were not detectable for our experimental task. These are influenced by geometry although in a way not detectable in our design due to tracing the path over several minutes rather than directly perceiving it in one glance.

Third, it may be that participants simply do not rely on any geometric features. Instead, it is possible that the small distance errors for the equilateral triangle type can be attributed to a working memory effect based on the equivalence of all three side distances. The overall systematic trends of overestimating angle and underestimating distance may have little to do with encoding geometric properties and instead be a result of execution errors [33, 59]. Overall, the lack of any consistent effects in angle and distance for specific triangle types in terms of accuracy and the lack of a correlation between angle and distance suggest, on a group level, that participants may not have been using triangle templates. The fact that triangle type does have separate yet significant influences on individual distance and angle errors leaves room for further investigation."

Medium comments:

R1.3) The section titles in the results seemed a bit too "conclusion-y" for a results section and seem better suited to the discussion (especially given the question about the conclusions in point 2 above). More descriptive section headers about the contents of the outcome measures would be appreciated.

We thank the reviewer for pointing this out. We have changed the section titles to be more objective and generic while still maintaining their purpose.

R1.4) For much of the results (as well as the figures), there is no mention of the statistical test used or what the design of the contrast is. It is also not listed in the methods. It seems like in some places there are portions of a 2-way ANOVA scattered around, but it was difficult to tell. Are these one-way tests against 0? Between triangle types? It was difficult to follow.

We apologize to the reviewer for a confusing presentation of the statistical tests used in our study. We have gone through and explicitly added the statistical test names and types.

R1.5) Throughout the results and methods, it was not entirely clear what the visual information was. Since the participants were wearing an HMD, it seemed at first like they would be seeing something (a ground plane?), but then when the comparison against the vision condition came up, I wasn't so sure. Was it primarily a blacked out screen for most of the trials? More details of the vision condition (maybe a figure of what people saw) would be helpful – was it just the monolith or was there anything else in the environment?

We apologize to the reviewer for the confusion. We have now added a figure of what participants saw to Supplementary Figure 2. During the "vision on" condition in Experiment #1, when participants reached the first monolith (G1), it would disappear and the next one would appear (G2). Once they reached that, it would disappear. There was no optic flow presented as we used the visual input as an additional condition in one experiment (#1) to ensure that participants were adequately encoding the stimulation provided by the hand-controlled. The HMD was used during the training of the treadmill to increase immersion and improve tracking.

Vision-on Condition

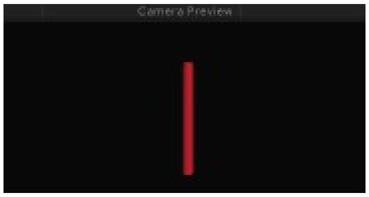

Vision-off Condition

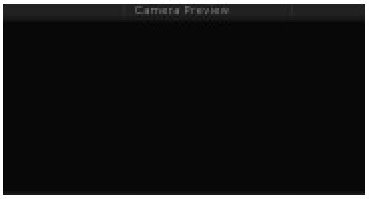

R1.6) It was not clear whether the number of trials (28 in Exp 1, 30 in Exp 2) was per triangle type or altogether. From the description of the trials in Exp 2 it looks like it might be altogether. That does not leave a lot of repeats for each triangle type (e.g. 4 in Exp 1), which could be tricky and underpowered to do the modeling. A few of the triangles in Exp 2 only had 2 trials, which is really difficult to be able to draw firm conclusions on – the authors note this themselves on page 20. Were the triangle types randomized or in a set order during the experiment? This is particularly important when it comes to modeling the history of previous trials.

We thank the reviewer for pointing this issue out. The low number of trials does increase difficulties in the modeling and model comparison, as shown in Supplementary Figure 7 & 9 in which we do not have ideal model recovery. However, the total trial numbers are similar to Loomis et al. 1993, and Klatzky et al. 1993 which we believe make for a reasonable grounds for comparison.

For trial order, we had 5 sets of pseudorandomized orders in which the same triangle type/size did not repeat in a row. There was no consistency between the pseudorandomized orders and each randomization set was randomly chosen for each subject.

For experiment 1, the table below shows each subject's trial order used

**Exp 1**				**Rand 1**	**Rand 2**	**Rand 3**	**Rand 4**	**Rand 5**
**SubjectID**	Trial Order			12	6	1	13	2
**AT02**	Rand 2			0	25	5	9	15
**AT03**	Rand 3			5	4	26	2	3
**AT05**	Rand 2		Total #	6	23	27	5	9
**AT07**	Rand 1	Rand 1	6	8	1	8	23	22
**AT09**	Rand 4	Rand 2	5	19	26	0	15	1
**AT10**	Rand 5	Rand 3	4	25	8	20	18	14

**AT11**	Rand 1	Rand 4	4	24	9	13	10	7
**AT12**	Rand 2	Rand 5	3	2	11	11	16	23
**AT13**	Rand 4			3	0	10	7	21
**AT14**	Rand 1			10	22	18	14	8
**AT15**	Rand 1			23	13	15	20	25
**AT16**	Rand 2		**Training**	11	3	9	22	10
**AT17**	Rand 3		30	22	17	24	4	13
**AT18**	Rand 4		33	17	2	22	8	18
**AT19**	Rand 3		31	7	27	25	12	5
**AT20**	Rand 5		32	15	14	16	21	12
**AT21**	Rand 5		28	16	19	3	6	0
**AT22**	Rand 4		29	26	16	14	25	26
**AT23**	Rand 1			21	12	21	0	11
**AT24**	Rand 1			4	24	7	3	27
**AT25**	Rand 2			27	5	6	19	20
**AT26**	Rand 3			20	10	2	1	19
				13	18	17	11	6
				18	21	23	26	16
				14	15	4	27	4
				9	20	19	24	17
				1	7	12	17	24

The number in the in the random sequences associated to the triangle index shown below is 0-13 for vision on conditions and 14-27 for vision off conditions. 30&33 are straight line trials.

Right Hand	∠bc	∠ab	∠ca	Side A	Side B	Side C
0&14	60	100	20	8.79385	3.47296	10
1&15	30	120	30	5.7735	5.7735	10
2&16	45	105	30	7.32051	5.17638	10
3&17	60	90	30	8.66025	5	10
4&18	60	75	45	8.96575	7.32051	10
5&19	45	90	45	7.07107	7.07107	10
6&20	60	60	60	10	10	10
Left Hand	∠bc	∠ab	∠ca	Side A	Side B	Side C
7&21	60	100	20	8.79385	3.47296	10
8&22	30	120	30	5.7735	5.7735	10
9&23	45	105	30	7.32051	5.17638	10
10&24	60	90	30	8.66025	5	10
11&25	60	75	45	8.96575	7.32051	10
12&26	45	90	45	7.07107	7.07107	10
13&27	60	60	60	10	10	10
Training	∠bc	∠ab	∠ca	Side A	Side B	Side C
28&31	30	130	20	6.52704	4.46476	10
29&32	45	115	20	7.80206	3.77377	10
30&33	0	0	0	20	0	50

For Experiment 2

**Exp 2**				**Rand 1**	**Rand 2**	**Rand 3**	**Rand 4**	**Rand 5**
**SubjectID**	**Trial Order**				9	8	6	5
**DT02**	Rand 2			5	2	3	1	6
**DT03**	Rand 3			6	1	5	6	1
**DT04**	Rand 4		Total #	1	5	2	5	3
**DT05**	Rand 3	Rand 1	3	2	8	7	2	1
**DT07**	Rand 1	Rand 2	4	8	6	5	7	6
**DT09**	Rand 5	Rand 3	4	7	0	8	5	8
**DT10**	Rand 3	Rand 4	4	0	1	7	1	2
**DT11**	Rand 1	Rand 5	2	6	5	0	8	7
**DT12**	Rand 2			4	2	1	6	4
**DT13**	Rand 4			3	9	6	9	3
**DT14**	Rand 1			5	7	2	8	7
**DT16**	Rand 4		**Training**	1	2	5	0	5
**DT17**	Rand 2		10	6	4	6	2	6
**DT18**	Rand 5		10	7	0	8	4	9
**DT19**	Rand 3		12	2	5	7	2	5
**DT20**	Rand 4		11	3	2	0	6	0
**DT21**	Rand 2		12	0	7	1	5	2
			10	5	5	6	7	5
				7	6	2	0	0
				0	1	1	7	1
				1	6	4	3	6
				7	0	3	1	7
				6	7	9	2	0
				2	3	5	7	5
				8	6	0	3	8
				1	7	7	0	7
				5	1	1	1	2
				2	0	0	6	1
				0	3	2	0	0

Again, the number in the Rand# are associated with left most column of the bottom table

Right Hand	∠ bc	∠ ab	∠ ca	Side A	Side B	Side C

0	40	80	60	5.276311	3.916222	6
1	40	80	60	8.793852	6.527036	10
2	40	80	60	43.96926	32.63518	50
3	40	80	60	87.93852	65.27036	100
4	40	80	60	175.877	130.5407	200
Left Hand	∠ bc	∠ ab	∠ ca	Side A	Side B	Side C
5	40	80	60	5.276311	3.916222	6
6	40	80	60	8.793852	6.527036	10
7	40	80	60	43.96926	32.63518	50
8	40	80	60	87.93852	65.27036	100
9	40	80	60	175.877	130.5407	200
Training	∠ bc	∠ ab	∠ ca	Side A	Side B	Side C
10	0	0	0	20	0	20
11	90	60	30	25	50	43.30127
12	60	60	60	10	10	10

R1.7) Based on the discussion on page 12 and elsewhere, the authors may want to consider a measure that combines distance and angle. One possibility is position error, just the straight-line distance the person was away from the target

We thank the reviewer for the suggestion and have created the plot below. It is now added it to supplementary materials as Supplementary Figure 5.

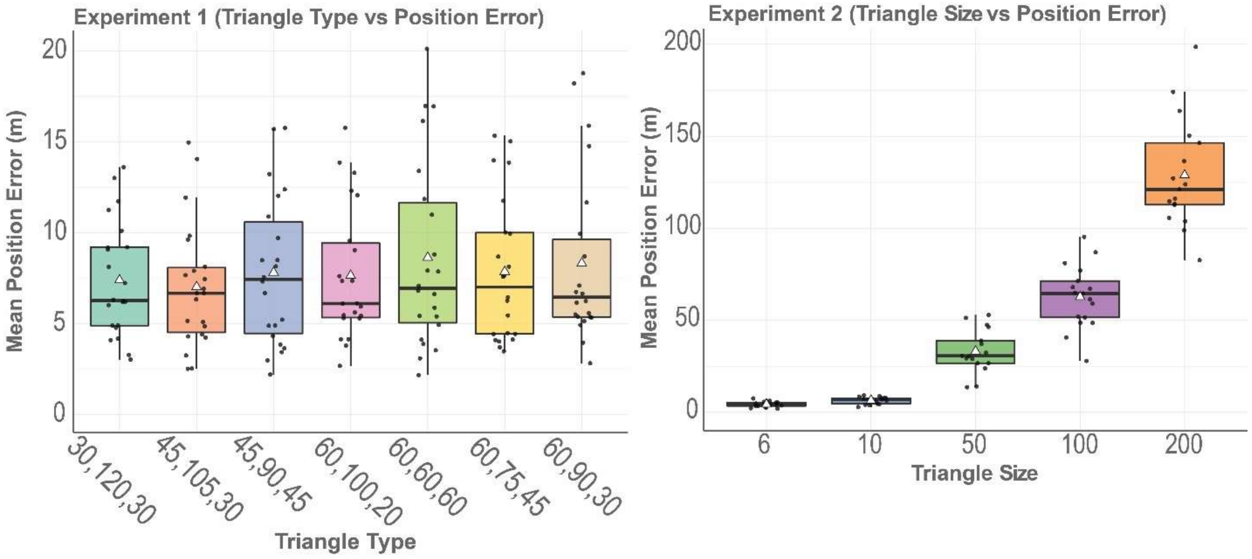

R1.8) I had a bit of difficulty following why the beta in line 392 was significant, but the larger beta from Exp 1 was not. Other people will probably wonder as well, so perhaps a bit of explanation about how the stats pan out here would be helpful.

We thank the reviewer for pointing this out. While redoing our modeling statistics we found a minor error and have updated the figure and the subsequent statistics. The history term is significant for both Experiment 1 and Experiment 2. We have made the appropriate change in the Result and Discussion section. We will be uploading the Matlab code along with our data to the GitHub repository.

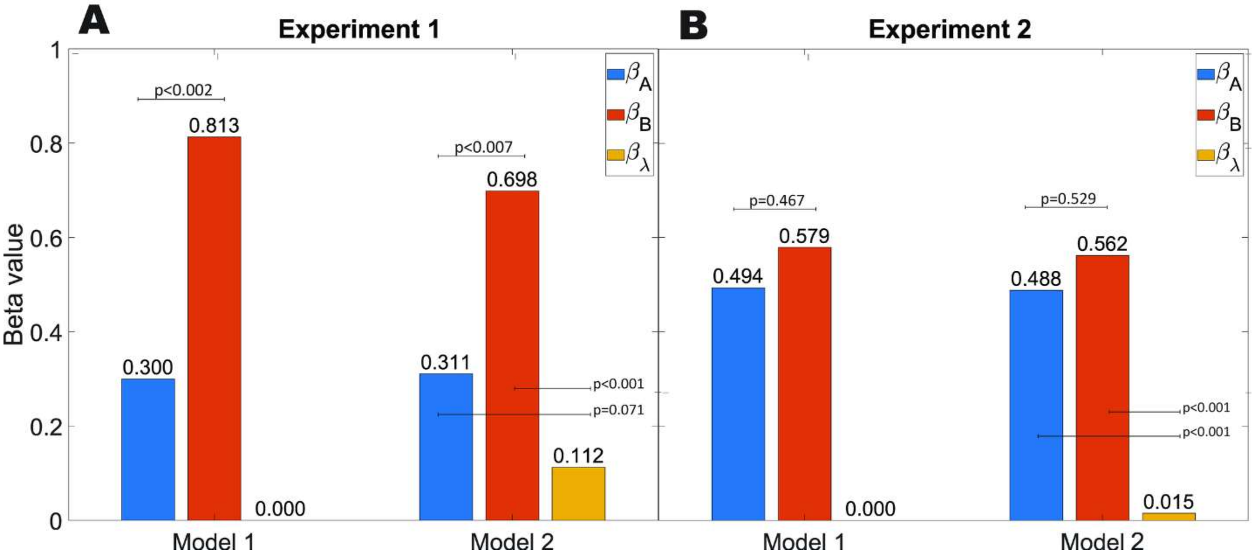

R1.9) The discussion on page 21 about the underestimation of the unguided leg with distance could be related to the execution of the homeward trajectory (assumption 4 of the Encoding-Error Model). A few people have looked at this idea, such as (Wan, Wang, & Crowell, 2013, Spatial Cog & Comp; Chrastil & Warren, 2017, Exp Brain Res).

We thank the reviewer for these important points. We have added the requested references and some discussion about execution error.

R1.10) For the claim on the last line of the main paragraph on page 22 (~line 471) seems like it needs more evidence to support it, especially given the distinction between homing vector and vector addition

We completely rewrote this paragraph to address reviewer's concerns and have removed that sentence.

"…Overall, the lack of any consistent effects in angle and distance for specific triangle types in terms of accuracy and the lack of a correlation between angle and distance suggest, on a group level, that participants may not have been using triangle templates. The fact that triangle type does have separate yet significant influences on individual distance and angle errors leaves room for further investigation."

R.11) The discussion about the difference between circuitous paths on the treadmill vs. the Souman study (page 24) is lacking a mention of a clear difference between the two: the treadmill included the feedback from the controllers to keep people going in a straight line. It is difficult to accept that the interface made no difference when the controller was providing information

We thank the reviewer for pointing this out. We note that the controller was only active for the encoded side of the triangle and not for the final (unguided side). Nonetheless, we agree with the reviewer that this could have been a factor even for the unguided side and after considering this paragraph in more detail, we believe it takes away from the main points of the paper and decided to remove it entirely.

R1.12) Limitations of the vector model: It wasn't clear what the authors meant by "directions", and perhaps this exposes where more clarity is needed about the vector model. Does the vector addition include the direction of the vector or just distance? If they do not have a direction, how are they a vector? Please clarify this aspect of the model.

We apologize to the reviewer for the lack of clarity. The vector addition model does include direction as x_A^t and x_B^t have both X and Y values. However, the β values scale both X and Y values equally hence the direction remain the same. The vector addition model scales sides A and B such that the origin and participant's final location becomes one. There are also individual noise components for both X and Y components, which can capture small variations in direction, but the model does not vastly rearrange the angle of sides A and B during fitting.

Below is a recreation Figure 1 in Fujita et al. 1993 (modified to employ the names of variable used in our study), as shown with the black solid line which represents the participant's walked trajectories. The dashed blue lines are the internal encoded representation as predicted by the Encoding-Error model (these are represented with subscript r). The dashed red line is a rough overlay for the paths predicted by the vector addition model. As shown below, the Encoding-Error model creates sides A_r and B_r and angle $\angle ab_r$. According to Euclidian properties, this results in side C_r=C and angle $\angle bc_{r=}\angle bc$. The vector addition model (in red), instead, adjusts A and B accordingly to fit participant's response C. Thus, the angle ∠aband ∠bc remain relatively the same in the vector addition model. The critical difference here is that the vector addition models assume a different suboptimal encoding of guided distance for sides A and B. In contrast, the Encoding-Error model assumes the same suboptimal encoding of the guided sides A B and separate suboptimal encoding of ∠ab such that it preserves Euclid's postulates.

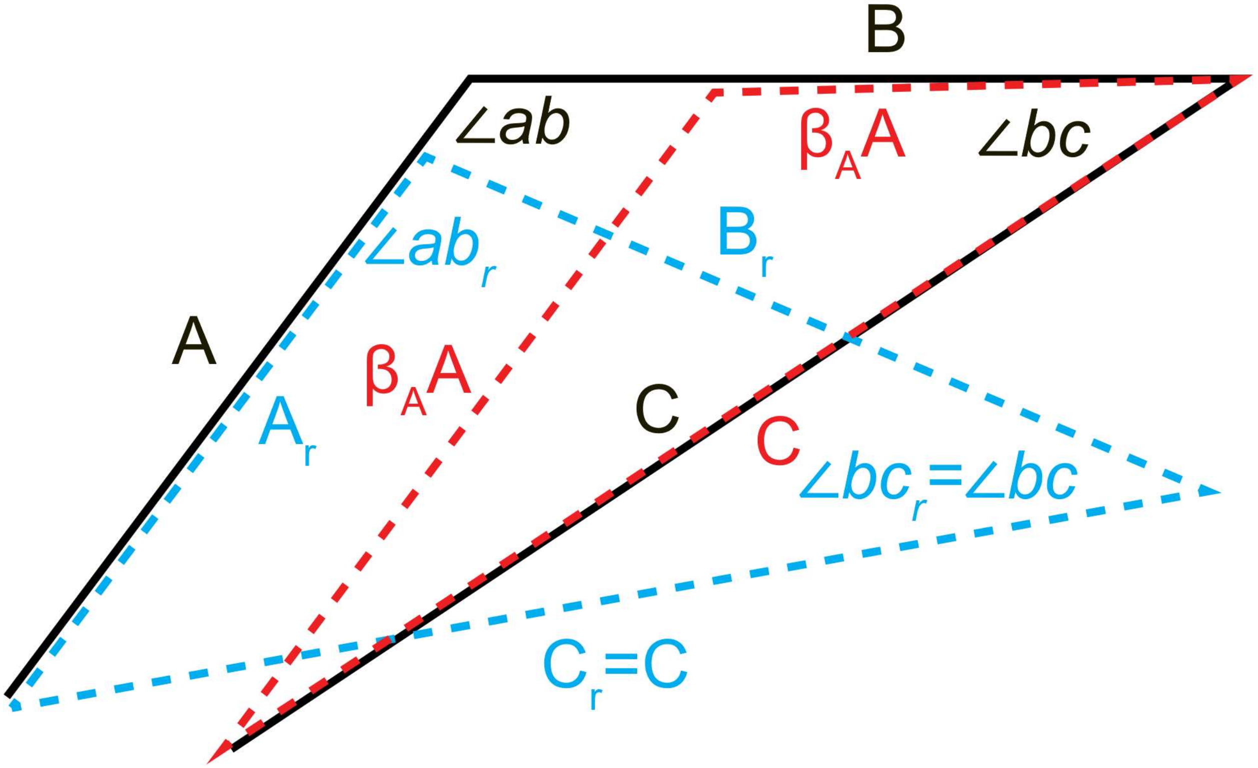

Note that this does not include fitting noise patterns which would slightly change the direction of all three sides. This would in fact make it non-Euclidean such that the total sum of all internal angles does not equal 180.

R1.13) A little more clarity in the modeling section of the methods would help, just to make sure it's clear what is in model 1 and what is in model 2. My reading is that model 1 is a weighted sum of the vectors, and model 2 is a weighted sum including noise whose variance is related to distance walked (on the outbound path?) and including a weighing of previous trials.

As the reviewer correctly states, both Model 1 and Model 2 contain a weighted sum of the vectors and noise components while Model 2 includes an additional term that differentially weights the contributions from previous trials. To be clear, the noise term, which scales with the distance of outbound path, is added when we simulate the models with the fitted β values. We added the follow text in the result section before the modeling results for clarification.

"…We employed two versions of a simple vector addition model to fit our results. Model 1 assigns weights to sides A and B and predicts the distance and direction of side C. (Equation 2). Model 2, in addition, includes a weighted influence of the participants' past trial history (Equation 5). We compared these two vector addition models to the Encoding-Error Model (Fujita et al., 1993)."

R1.14) Figure 2: Explain what the statistical tests are and what they show (e.g. ANOVA examining differences between triangle types, and a significant effect of

triangle type was found). Figure 3 could use more information in the caption. Figures 4-6: It might be clearer to arrange by Experiment rather than by model, so that it becomes 2 figures (one for Exp 1 and one for Exp 2). It is easier to compare the three models within an Experiment. Perhaps also show the actual data in that combined figure.

We thank the reviewer for the suggestion and have rearranged the Figures, as shown below. We have also modified the caption in a manner consistent with the reviewer suggestions.

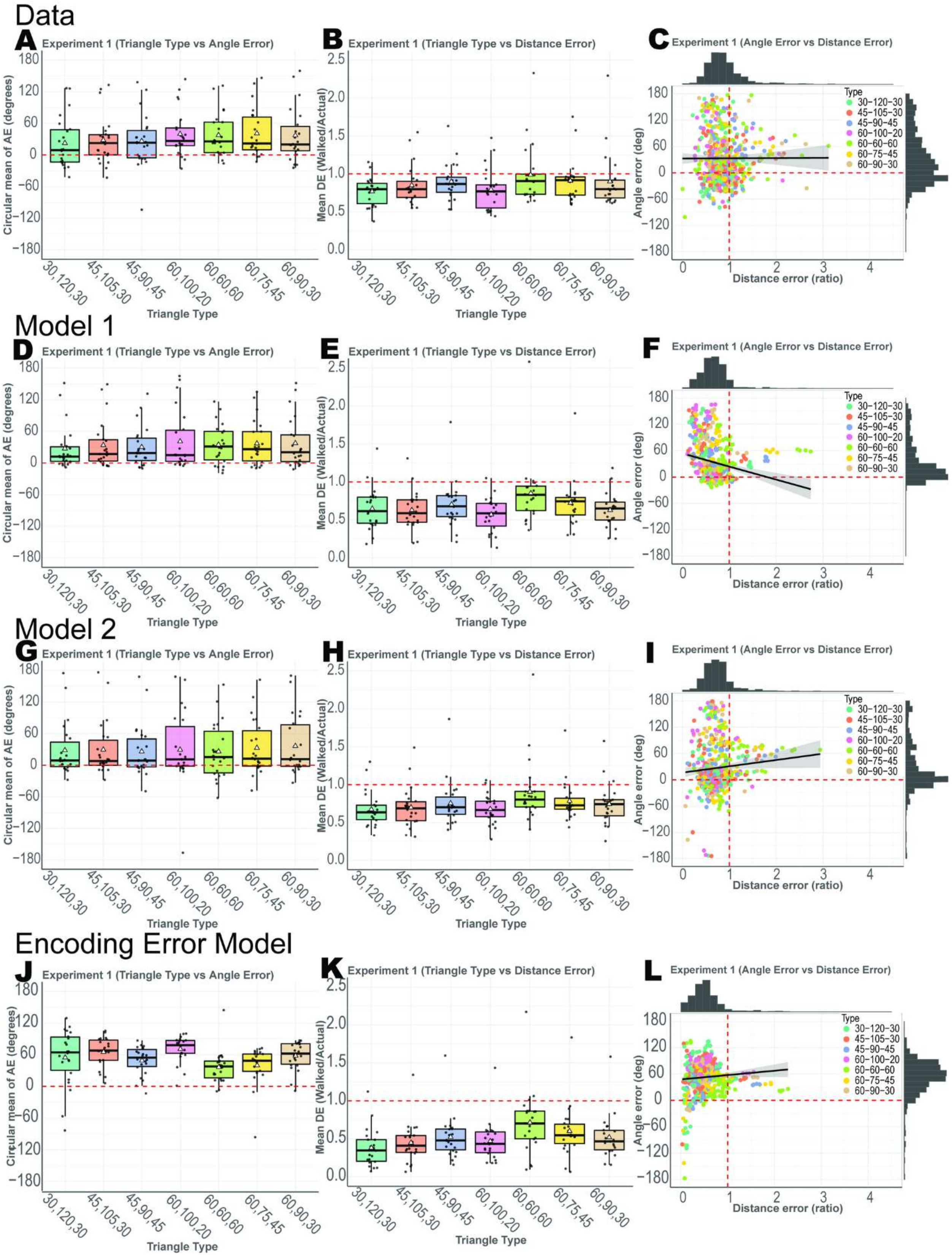
Data
A
Experiment 1 (Triangle Type vs Angle Error)
Circular mean of AE (degrees)
Triangle Type
B
Experiment 1 (Triangle Type vs Distance Error)
Mean DE (Walked/Actual)
Triangle Type
C
Experiment 1 (Angle Error vs Distance Error)
Angle error (deg)
Distance error (ratio)
Type
30-120-30
45-105-30
45-90-45
60-100-20
60-60-60
60-75-45
60-90-30
30,120,30
45,105,30
45,90,45
60,100,20
60,60,60
60,75,45
60,90,30
Model 1
D
Experiment 1 (Triangle Type vs Angle Error)
E
Experiment 1 (Triangle Type vs Distance Error)
F
Experiment 1 (Angle Error vs Distance Error)
Model 2
G
Experiment 1 (Triangle Type vs Angle Error)
H
Experiment 1 (Triangle Type vs Distance Error)
I
Experiment 1 (Angle Error vs Distance Error)
Encoding Error Model
J
Experiment 1 (Triangle Type vs Angle Error)
K
Experiment 1 (Triangle Type vs Distance Error)
L
Experiment 1 (Angle Error vs Distance Error)

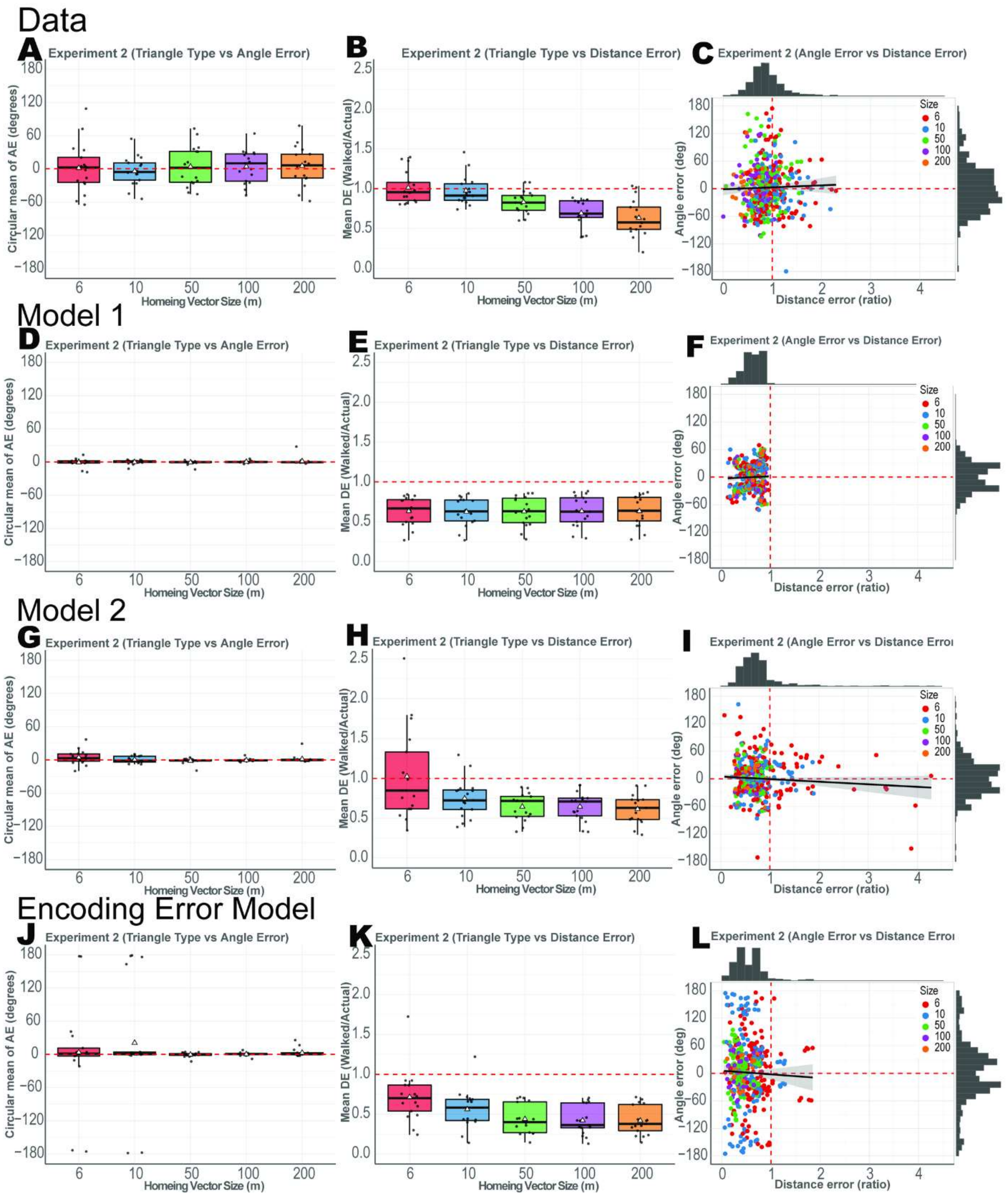

R1.15) In the supplement, S6 seems like it might contract the findings in the results. It is also a bit confusing about the difference between figures S6 and S8, as well as S9. Why is model 1 removed here?

We apologize for the confusion in our model comparison figures. Supplementary Figure 6 A&D shows that Model 2 best fits most participants' behavior. However, when we use AIC and BIC (Supplementary Figure 6 B, C,E, and F) which penalize the models for the number of parameters we see Model 1 performs the best. In Supplementary Figure 7, we employ a method of model recovery, which is used to further validate the model comparisons. Because we do not see diagonal values close to 1 (meaning model X's simulation data best fits itself), we are unable to reach firm conclusions from the results in Supplementary Figure 6 B, C, E, and F.

We chose to remove Model 1 in Supplementary Figure 8&9 as we thought the low number of trials and the similarity between Model 1 and Model 2 may have resulted in poor model recovery. While the diagonal values improved in Supplementary Figure 9, it is not to a level where we can reach firm conclusions from the results in Supplementary Figure 8.

While our model recovery is poor, the maximum likelihood fits (Supplementary Figure 6 A&D and Supplementary Figure 8 A) show the Vector Addition models as the better fit even with the Encoding-Error Model having more parameters. This suggests that Vector Addition models may be more accurate and parsimonious, at least for this dataset.

R1.16) Appendix A needs some additional clarity. Define b1, b3, and b4. Presumably these are the parameters you are fitting, but make it explicit. Explain why the same beta number (e.g. b3) appear in multiple equations. Explain more clearly what Level 1 and Level 2 are modeling, it currently doesn't quite line up to be clear. What values are taken from the experimental design (such as the length of the outbound legs) and what values are taken from the data?

We apologize to the reviewer for not clarifying out modeling approach. We have added more description to the in the Appendix section. Please see our changes in Appendix A.

Minor comments:

R1.17) Line 150, "pathway" should be "pathways"

We apologize for this error and have made the suggested change.

R1.18) Line 382, add that the beta values were significantly less than 1. (Was this a 1- sample t-test against 1?)

We apologize for the confusion we have added more details for all statistical tests. Additionally, the reviewer is correct: we employed a 1- sample t-test against 1.

R1.19) Line 562, "…plausible then…" should be "…plausible than…"

We have made the suggested change.

R1.20) Line 652, how did the controller provide feedback? Vibrate on the side to turn towards or turn away from?

To address this issue, we have added the following text:

"…for example, if the right controller vibrated, they would need to turn left"

R1.21) I suggest moving the remainder of the experimental methods before the modeling, because it seems to kind of break up the flow of the analysis.

We thank the reviewer for the suggestion. We are, however, following the manuscript organization structure suggested by PloS (https://journals.plos.org/ploscompbiol/s/submission-guidelines)

If this structure is not required, we are happy to implement the reviewer's suggestion.

R1.22) In the modeling portion, there are a few places where the notation was not clear. When first introduced, xt c does not explain what the t superscript is – it comes out eventually in a few pages but could use at least a mention early on that it is the current trial. Later on, χ looks a lot like x (it took me a while to realize they were different), so you might want to consider a different symbol. Similarly, a and b were used in the description of the triangles (e.g. Figure 1), but were also used in the modeling portion to refer to something different. This could create confusion.

We apologize to the reviewer for the similar-looking variable names. We have changed $\boldsymbol{\chi}$ to λ as well as defining the t superscript when first shown. Additionally ,we changed angle $\boldsymbol{\alpha}$ to $\angle ab$ and $\boldsymbol{\beta}$ to $\angle bc$.

Reviewer #2

Major comments:

R2.1) I was possibly having a particularly dense moment, but I was at page 11 of the paper when I finally realized that the terms ‘guided leg’ and ‘unguided leg’ did not mean the participant’s legs but the epochs of the experiment. I very much dislike the use of the term ‘leg’, especially in a walking experiment. Instead, I would suggest using epoch, phase, portion, side (of triangle) or some other similar term.

We understand this confusion. We replaced the term 'leg' with 'side' throughout the entire paper to avoid this confusion.

R2.2) In the abstract and at a couple of places in the manuscript, you mention that the distances were kept ‘relatively constant’ in experiment 1, and angles were kept ‘relatively constant’ in experiment2. Why were these only relatively constant and not designed to be exactly alike? Are you referring to the participants’ actual trajectory in the guided portion, which slightly differs from the ideal trajectory? If so, that was not clear to me as I read the paper.

We apologize to the reviewer for the confusing term ‘relatively constant’ which was meant to refer to participants' actual imperfect trajectories. To clarify, we kept the return distance for each triangle exactly constant in Experiment 1, doing so by ensuring the distance of the guided sides for each triangle differed (Supplementary Table 1). We changed the ‘relatively constant’ term in the paper to ‘constant’ and provided the following text to clarify this issue in the procedure of Experiment 1.

“…To keep side C’ at a constant 10m across all 7 triangles, we employed different side A’ and side B’ (guided portion) sizes to accommodate the different angles.”

R2.3) Similarly, lines 338-339. “...maintained a scalene triangle shape, thus leaving angle as comparatively constant as possible”. Why comparatively? It should be possible to literally scale the same triangle to get whatever distance required, maintaining exactly the same angle.

We again apologize to the reviewer for the term “comparatively constant.” The term “comparatively” was meant to accommodate the idea that actual participant trajectories might differ slightly. To be clear, in Experiment 2, we did indeed keep the triangle angles exactly constant across different triangle sizes. We have edited the sentence from “...maintained a scalene triangle shape, thus leaving angle as comparatively constant as possible” to “...maintained a scalene triangle shape, thus leaving angle constant.”

R2.4) There is some conflation between the terms 'homing' and 'path integration' in the paper. Homing is the behavior, and path integration is the computation that could potentially result in homing. It would be worth mentioning this difference in the manuscript.

We apologize for the conflation between terms for homing (behavior) and path integration (computation). This issue was raised by both reviewers and we have gone to great lengths to provide clarification and draw distinctions between the two.

First, we have now tried to be clearer about the theoretical differences between Encoding-Error and vector addition models. We now clarify and setup the theoretical differences between continuous models and configural models in significantly more detail. To avoid confusion, we now refer to homing vector created in continuous models as a "continuous homing vector" and homing vector created in configural models as "configural homing vector."

"Past experiments on path integration have often involved a path completion task in which the navigator is guided in physical space and must return using the shortest trajectory back to the origin with no guidance [2, 21,22]. Behavioral results both in vertebrates and invertebrates show a systematic bias in the return trajectory which is independent of random accumulated noise [23-27]. Two sets of computational models have been proposed as potential strategies participants might use, in some form, for path integration. The Homing Vector Model (also called a continuous strategy) assumes that the navigator does not encode information about the outbound path but rather continuously updates their current position relative to the origin by computing a continuous homing vector. To capture systematic errors in return paths, Homing Vector Models assume different variations of memory decay or leaky integration which are in theory independent of outbound path configurations [28-30].

Configural Models, in contrast, suggest a continuous encoding of the outbound paths (guided path) which are then manipulated using trigonometric or vector calculations to compute a return trajectory (configural homing vector) when required. One of these models, the Encoding-Error Model, suggests that systematic errors in the return path are due to errors in encoding of the outbound paths and their relative configuration [31, 32]. Recent work, however, suggests that execution errors (during the unguided path) make the largest contribution to systematic errors [33]. Furthermore, Wiener et. al. systematically compared configural and continuous updating strategies by explicitly telling participants to focus on the outbound path or the origin. The results suggest that participants can deploy either configural or continual strategies during a path completion task [34]. Given the widespread application of configural models to both human and non-human path integration [23, 24, 26, 31, 32, 35] and the evidence that humans employ both configural and homing strategies [34], we directly compared three different configural models of path integration to better understand the patterns of errors that accumulate as participants dead-reckon."

Second, we made sure to explicitly mention configural or continuous when we use the term ‘homing vector’ to signify whether we mean a computed return vector or an integrated one.

R2.5) Line 100-106. Here you introduce the Encoding-Error Model for the first time, and state the assumptions of the models, but yet do not state what the model actually does. I think a brief description of the model is warranted here, perhaps similar to what you later state in the Discussion, lines 566-568.

We thank the reviewer for pointing this out. We have added the following text in the introduction as suggested.

“…This model uses the law of cosines to compute the third side of the triangle given the distance and angles encoded during guided portions.”

R2.6) Lines 251-264: Why does the triangle type need to have an effect on both angle and distance errors for it to be deemed significant? You do mention elsewhere that it is possible that some sensory modalities have more influence on linear vs. angular motion estimates. Indeed, in these experiments, distances are more likely underestimated and angles are more likely overestimated. I am unsure why the low effect size on angle errors discounts the result of the effect of triangle type on distance errors. Also, extending the same logic, wouldn’t size of the triangle be insignificant since it did not have an effect on both angle and distance errors?

We thank the reviewer for bringing up this point. We have changed the Results section to be more objective and focused on the statistics. Additionally, we have changed the discussion section to reflect on the statistics and offer 3 plausible explanations for our findings.

Results section for Experiment 1
".... While we did not find a consistent effect of triangle type across angle and distance errors, it is clear the triangle type contributed significantly to the patterns of participant errors. For example, the isosceles triangle (30,120,30) showed the lowest mean angle error (10.96°±9.11°) yet the equilateral triangle demonstrated the lowest mean distance error (0.985±0.062). Whether participants were relying on triangle templates, abstract geometric cues, or instead repeating identical/similar past paths in working memory, is an issue we return to in the Discussion… Together, these findings suggest that while geometric features (angle and distance) contribute to participant errors, it is not clear how these features are combined (or if at all) to create and deploy triangle templates."

Discussion section Experiment 1

" In Experiment 1, we found that the triangle type tested had an influence on participants' distance and angle errors on the unguided side. We found that the equilateral triangles showed significantly lower distance error and similarly, we found a weak tendency for the isosceles triangle angles (30,120,30) to show lower angle overestimation. All triangle types observed in Experiment 1, however, showed an angle overestimation and distance underestimation. The question of whether the differences in distance and angle errors observed are due to participants using triangle templates is one we sought to answer in this study.

We believe that If participants were using a triangle template, then we would be able to observe accurate distance and angle estimations for one triangle type. For example, it might be possible to predict that equilateral triangles or right triangles would be overall more accurate than scalene triangles. This is because these geometries are far more regular and potentially easier to encode holistically, particularly given their influence on visually-guided navigation [53]. While we did not find one triangle type to have lower distance and angle errors at the group level, there may be several explanations for how triangle type had an influence on participants' performance.

First, we found two participants who showed a high degree of accuracy for both distance and angle on equilateral and right isosceles triangles (Supplementary Figure 4C). This suggests that some participants may have used a strategy that involved deploying templates. Unlike Wiener et al. 2011 [34], we did not explicitly ask participants to focus on the outbound path or the origin, which in turn could possibly have affected some participants' likelihood of spontaneous using a template. Our results, in this way, build on other studies showing individual differences in human navigation studies [54-56].

A second explanation for how triangle type may have influenced participants' performance focuses on the possibility of using abstract geometric features. Studies in young children suggest that rather than using room geometry to navigate, children use certain geometric features (i.e. the distance between walls) to reorient themselves [57, 58]. In our task, participants were not directly exposed to the geometric regularities of the path but rather they had to "trace" it by walking it. This process may conserve some geometric features and not others, which may in turn influence the pattern of errors. In particular, because participants could not directly perceive the entire shape, they may have attempted to guess at it, and if their memory for a side was inaccurate, this would affect their memory for the entire shape. Thus, while we could not determine any correlation between patterns of errors and any obvious geometric features (triangles with an acute or obtuse and right angle), there may be other features that were not detectable for our experimental task. These are influenced by geometry although in a way not detectable in our design due to tracing the path over several minutes rather than directly perceiving it in one glance.

Third, it may be that participants simply do not rely on any geometric features. Instead, it is possible that the small distance errors for the equilateral triangle type can be attributed to a working memory effect based on the equivalence of all three side

distances. The overall systematic trends of overestimating angle and underestimating distance may have little to do with encoding geometric properties and instead be a result of execution errors [33, 59]. Overall, the lack of any consistent effects in angle and distance for specific triangle types in terms of accuracy and the lack of a correlation between angle and distance suggest, on a group level, that participants may not have been using triangle templates. The fact that triangle type does have separate yet significant influences on individual distance and angle errors leaves room for further investigation."

R2.7) Page 14: I think that the models need to be more clearly enumerated. Model 2 is mentioned (Line 294) before Model 1 is, even though model 1 is what you are talking about in the previous paragraph. Suggestion: "We employed two versions of a simple vector addition model to fit our results. Model 1 assigns weights to legs A and B, and predicts the distance and direction of leg C. (Equation 2). Model 2, in addition, includes a weighted influence of the participants' past trial history (Equation 5). We compared these two vector addition models to the Encoding-Error Model (Fujita et al., 1993)."

We thank the reviewer for pointing this out and have added the suggested text before mentioning results from Model 1.

"We employed two versions of a simple vector addition model to fit our results. Model 1 assigns weights to sides A and B and predicts the distance and direction of side C (Equation 5). Model 2, in addition, includes a weighted influence of the participants' past trial history (Equation 6). We compared these two vector addition models to the Encoding-Error Model [31]."

R2.8) You use the variables α and β both to refer to parameters in the vector addition models and also angles between the legs in the task. I would suggest using different variables to maintain consistency and clarity.

We apologize to the reviewer for multiple uses of **α** and **β**. We changed angle **α** to ∠ab and **β** to ∠bc.

R2.9) One key way in which your experimental setup differs from real-world walking is the lack of linear vestibular cues. This is an unavoidable trade-off of being on an omnidirectional treadmill, but I feel like this needs to be explicitly stated and discussed somewhere in Pages 24-25. For example, you could state that desktop VR preserves optic flow, but lacks other path integrative cues. VR with a treadmill would have proprioceptive and motor efference copy signals, but not vestibular cues. VR with omnidirectional treadmill will still lack linear vestibular cues, but preserve angular vestibular cues.

We thank the reviewer for pointing this out. VR on an omnidirectional treadmill does indeed involve the full-range of vestibular rotational cues as participants physically turn their body in the treadmill. With regard to translational vestibular cues, participants also do tilt slightly forward as they walk although the net displacement is zero. Thus, we do think that the omnidirectional treadmill involves contributions from both vestibular systems, just not to the same extent as real-world navigation. We have added the following text in the suggested discussion paragraph.

“The treadmill, however, has diminished translational vestibular cues although it is notable that the distance underestimation results we obtained were largely comparable to past studies using the real-world triangle completion task [32]. As such, we were able to capture novel aspects of non-visual navigation otherwise difficult to observe in desktop-VR.”

R2.10) In lines 573-574, you mention that the parameters of the linear functions of the Encoding-Error Model may not generalize across participants. However, why would the linear relationship between the guided leg and the encoded vector in the vector addition model generalize across participants? In general, I don’t think parameter generalization across participants is necessary for a good model, the model structure being preserved and having predictive power across participants is more important

We thank the reviewer for pointing this out and after reviewing it we agree with the reviewer and have taken out the text regarding parameter generalization across individuals.

R2.11) Line 682: “Combining the first two suboptimalities…” which are the two? Only one suboptimality (over or under-weighting) is mentioned before this. I assume you mean noise as the other, but that is not mentioned.

We apologize to the reviewer for the confusing text. By ‘suboptimalities’ we meant the suboptimal encoding of side A and side B (i.e. $\beta_A x_A^t$ and $\beta_B x_B^t$). However, we changed ‘suboptimalities’ to ‘side’ to have better consistency across the entire paper.

R2.12) I am confused by Equation 3. Why is the noise centered around xC t? There is almost always a consistent bias of underestimating distance, and so sampling around the optimal vector xC t should not be correct. Do you intend to say that you sampled around the mean vector for each participant?

We thank the reviewer for pointing this out. We realize our equation had errors and have updated this section accordingly.

Equation 3 is centered around $\tilde{x}_D^t$

$$3.\quad P(x_D^t \,|\tilde{\sigma}, \beta_A, \beta_B) = \frac{1}{\sqrt{2\pi\sigma^2}} exp(-\frac{(x_D^t - \tilde{x}_D^t)^2}{2\sigma^2})$$

where

$$\tilde{x}_D^t = -(\beta_A x_A^t + \beta_B x_B^t)$$

And

$$x_D^t = -(\beta_A x_A^t + \beta_B x_B^t) + \varepsilon$$

Where ε is the noise term sampled from a normal distribution with mean 0 and standard deviation σ.

R2.13) χ and x are very similar-looking symbols and make Eqns. 5 and 6 much less clear. Please use another variable instead of χ to denote linear combination of past vectors.

We apologize to the reviewer for the similar-looking variable names. We have changed **χ** to λ.

R2.14) What is β in Eqn. 7? I assume it is [βA , βB , βχ], but please list that explicitly

We apologize to the reviewer and have clarified by explicitly listing each $\boldsymbol{\beta}$ as suggested.

R2.15) I would suggest using something other than the white triangles to represent mean in Figs. 2, 4, 5, 6. The marker is low-contrast and not easily visible.

We apologize to the reviewer for the low contrast indicators for mean values in Figs. 2, 4, 5, 6. We have added a black outline around white outline, which we hope improves the visibility.

R2.16) Why are the angle errors so tightly clustered in the simulated model data? Figs. 4D, 5D, 6D

We thank the reviewer for pointing this out. The values (i.e. individual points) in figures are mean values of each subject (mean angle error of all 60-60-60 for subject X). We believe this is the cause of the clustering.

R2.17) Figures need more formatting and consistency in font sizes. E.g.

a. **Subfigure names A-H are too large in Figure 1**
b. **Fig 1. C,D are clearly horizontally squished.**
c. **Labels G1, G2 etc. need to be separated from the markers in Fig. 1 G, H**
d. **Axes labels and legends need to be larger and consistently sized, e.g. in Figs. 1G, 1H, 2, 4, 5, 6**
e. **Units of quantities in axes labels, e.g. Figs. 1G, H**

We thank the reviewer for the feedback have made the suggested change.

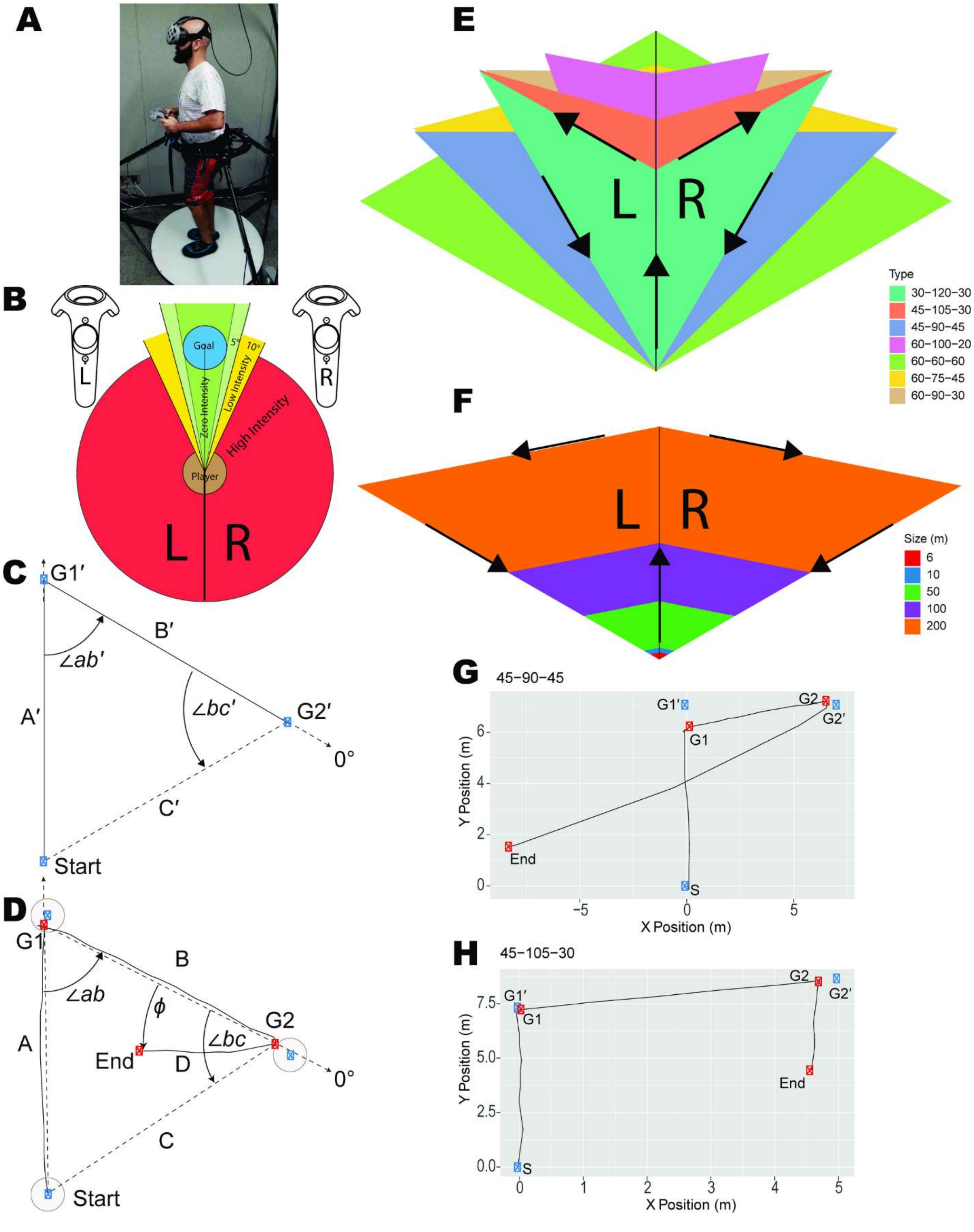

Minor comments:

R2.19) Line 73: Path integration in cats does not have a citation.

We apologize for this error and have made the suggested change.

R2.20) Line 76-77: "research on path integration has often been neglected in favor of situations in which visual input provides sufficient information to solve most navigational tasks, such as in desktop virtual reality." I think it should be mentioned here that vision is not completely lacking in path integrative cues. Optic flow provides a strong velocity cue which can be used for path integration. However, in the real world, the effects of visual landmarks and optic flow cannot be easily decoupled. Virtual reality is one of the ways of accomplishing this.

We thank the reviewer for clarifying this we have added the following text:

*"*Because humans employ vision as a primary modality to navigate, research on path integration has often focused on situations in which visual input provides sufficient information to solve most navigational tasks, such as in desktop virtual reality. This is because visually-rendered optic flow provides a velocity signal sufficient for some forms of path integration, with desktop virtual reality providing the opportunity to decouple visual from body-based cues in freely navigating humans. A limitation, however, with desktop VR is that it lacks the enriched cues and codes that we obtain when we freely move our body throughout space, thought to contribute to critically to path integration [18-20], and does not allow comparison of the effects of visual vs. body-based input on path integration..*"*

R2.21) Line 90:I would suggest using the term linear algebra instead of matrix algebra. The properties that you mention are linear transformations, matrices are simply a way of representing them and manipulating them.

We have made major changes to this paragraph and have removed this this sentence.

"…we created a simple yet novel configural model based on vector addition often used to understand path integration in other species [23, 24]. The model assumes the outbound path is encoded as discrete sets of vectors, such that individual vectors can be used to compute new trajectories using as a configural homing vector..."

R2.22) Line 97: "and then must return" -> "and then the participant must return"

Thank you. We have made the suggested change.

R2.23) Line 111: "...paths that crossed each other included in Klatzky et al. 1990" -> "...paths that crossed each other."

We have made the suggested change.

R2.24) Line 150: pathway -> pathways

We have made the suggested change.

R2.25) Line 156: What do you mean by "single value encoding” here? The terminology is used but never explained.

We have changed the term to “configural encoding”

R2.26) Line 165: infinite large spaces -> infinitely large spaces.

We have made the suggested change.

R2.27) Line 167: over shoot -> overshoot

We have made the suggested change.

R2.28) Line 185: set-up -> setup

We have made the suggested change.

R2.29) Line 209: participants -> participants’

We made the suggested change.

R2.30) Line 212: 0.87 +/- 0.5, which was, on average, close to the the correct response of 1. This is a very qualitative statement. What does the term ‘on average’ mean here? It is already stated that 0.87 is the average value.

We apologize for this error, we did not mean to say ‘on average.’ We have removed the word “average” accordingly.

R2.31) Line 236: little effect the direction -> little effect on the direction
Lines 324-326: The sentence structure is confusing. Please clarify.
Line 423-424: 5 subject data -> 5 subject’s data.

We apologize for these errors and have made the suggested changes.

R2.32) Line 638: controller -> controllers’

We made the suggested change.

R2.33) Hand-held vs. handheld. I am not sure which one is more accurate, but please stick to one.

“Handheld” appear correct. We have made that change.

R2.34) Line 698: xC t should be xD t

We have have made the suggested change.

R3.35) Line 759: templets -> templates

We have made the suggested change.

R3.36) Line 791: the15m -> the 15 m

We have made the suggested change.

R3.37) Consistency in citations. In various places you have “Author et al., 2000”, “Author et al. 2000”, “Author1 & Author2 2000”, “Author1, Author2, Author 3, & Author 4, 2000” etc. Please check the style guide for PLOS and format citations according to that.

We apologize for these errors and have made the suggested change. We now use PLoS formatting for citations which involves numbers.

R3.38) Fig. 1 caption: The blue denotes -> the blue point denotes

We have made the suggested change.

R3.40) Fig 1F caption: templets -> templates, experiment 1 -> experiment 2

We have made the suggested change.
