# Supplementary material for: Path integration in large-scale space and with novel geometries: Comparing vector addition and encoding-error models
Source: PLoS Comput Biol. 2020 May 7;16(5):e1007489. doi: 10.1371/journal.pcbi.1007489 (PMC7244182; doi:10.1371/journal.pcbi.1007489)
Supplement: Attachment — Submitted filename: plosCompBiol2019_rev2.docx [file pcbi.1007489.s017.docx]

Path Integration in Large-Scale Space and with Novel Geometries: Comparing Vector Addition and Encoding-Error Models

Reviewer Comments – second round

**Notes to authors**

Thank you for thoroughly and sufficiently addressing my initial round of comments. The story is much more coherent and clear now. I am especially pleased with your modifications to the terminology and descriptions of configural vectors, homing, and the computation of path integration. I am also happy with the modifications to the model equations – there were no obvious errors or required clarifications.

Some minor comments follow:

(1) Line 27: “limited insight into their contributions” -> “limited insight into their respective contributions”
(2) Lines 56-58: “We found no effect of triangle shape on systematic errors, while path length scaled the systematic errors logarithmically, similar to Weber-Fechner law.” Suggestion: “We found no effect of triangle shape on systematic errors, while the systematic errors scaled with path length logarithmically, similar to Weber-Fechner law.”
(3) Line 59: participant’s -> participants’
(4) Line 66-70: Suggest inverting the sentence structure. “All animals species tested, including <list of animals> show the ability to dead reckon, thought to involve the computational process called path integration.”
(5) Lines 71-72: “focused on situations” -> “focused on experiments”
(6) Lines 77-79: “thought to contribute to critically to path integration, and does not allow comparison of the effects of visual vs. body-based input on path integration.” Suggestion: “thought to contribute critically to path integration, and does not allow comparison of the effects of visual vs. body-based inputs on path integration.”
(7) Line 96: configuration -> configurations
(8) Line 111: “the triangle completion task more specifically” -> “specifically the triangle completion task”
(9) Line 113: distance -> distances
(10) Line 129: small sized environments -> small environments
(11) Line 148: (equilateral triangles) -> (such as equilateral triangles)
(12) Line 169: treadmill is it permits -> treadmill is that it permits
(13) Line 181: using as a configural -> using a configural
(14) Line 184: “Here, we set out to” -> “In Experiment 1, we set out to”. Also delete “in Experiment 1” on Line 187.
(15) Lines 206-209: “As can be seen in the raw example shown (Figure 1G & H) and others (Supplementary Figure 1), although participants were often quite accurate at completing the triangle, they tended to overestimate the angle and underestimate the distance, regardless of triangle type”. However, Figures 1G and 1H are examples where participants under- or over-estimate both angle and distance. Maybe refer to Figs 2A and 2B instead.
(16) Line 210: specifically, with Klatzky et al. -> specifically with Klatzky et al.
(17) Lines 223-224: This sentence is unnecessary. 0.87+/-0.5 does not seem that close to 1. I would suggest that it be simply "The normalized walked distance was 0.87 +/- 0.5”.
(18) Lines 279-280: Parentheses around stats
(19) Line 326: Delete “as well”
(20) Line 358: leaving angle constant -> leaving the angles constant
(21) Lines 384-386: Very confusing sentence structure.
(22) Line 401: model -> models
(23) Lines 451,452: subject’s -> subjects’
(24) Line 510: guess at it -> guess it
(25) Line 512-513: Delete phrase in parentheses. I don’t think it is necessary.
(26) Line 578: participant’s -> participants’
(27) Line 583: subjects -> participants
(28) Line 622: Experiment #1 -> Experiment 1
