# Supplementary material for: Path integration in large-scale space and with novel geometries: Comparing vector addition and encoding-error models
Source: PLoS Comput Biol. 2020 May 7;16(5):e1007489. doi: 10.1371/journal.pcbi.1007489 (PMC7244182; doi:10.1371/journal.pcbi.1007489)
Supplement: Attachment — Submitted filename: plos_rev2.docx [file pcbi.1007489.s018.docx]

Some minor comments follow:

(1) Line 27: “limited insight into their contributions” -> “limited insight into their respective contributions”

*We have implemented this suggestion.*

(2) Lines 56-58: “We found no effect of triangle shape on systematic errors, while path length scaled the systematic errors logarithmically, similar to Weber-Fechner law.” Suggestion: “We found no effect of triangle shape on systematic errors, while the systematic errors scaled with path length logarithmically, similar to Weber-Fechner law.”

*We have implemented this suggestion.*

(3) Line 59: participant’s -> participants’

*We have implemented this suggestion.*

(4) Line 66-70: Suggest inverting the sentence structure. “All animals species tested, including <list of animals> show the ability to dead reckon, thought to involve the computational process called path integration.”

*We have implemented this suggestion.*

(5) Lines 71-72: “focused on situations” -> “focused on experiments”

*We have implemented this suggestion.*

(6) Lines 77-79: “thought to contribute to critically to path integration, and does not allow comparison of the effects of visual vs. body-based input on path integration.” Suggestion: “thought to contribute critically to path integration, and does not allow comparison of the effects of visual vs. body-based inputs on path integration.”

*We have implemented this suggestion comment.*

(7) Line 96: configuration -> configurations

*We have implemented this suggestion.*

(8) Line 111: “the triangle completion task more specifically” -> “specifically the triangle completion task”
(9) Line 113: distance -> distances

*We have implemented this suggestion.*

(10) Line 129: small sized environments -> small environments

*We have implemented this suggestion.*

(11) Line 148: (equilateral triangles) -> (such as equilateral triangles)

*We have implemented this suggestion.*

(12) Line 169: treadmill is it permits -> treadmill is that it permits

*We have implemented this suggestion.*

(13) Line 181: using as a configural -> using a configural

*We have implemented this suggestion.*

(14) Line 184: “Here, we set out to” -> “In Experiment 1, we set out to”. Also delete “in Experiment 1” on Line 187.

*We have implemented this suggestion.*

(15) Lines 206-209: “As can be seen in the raw example shown (Figure 1G & H) and others (Supplementary Figure 1), although participants were often quite accurate at completing the triangle, they tended to overestimate the angle and underestimate the distance, regardless of triangle type”. However, Figures 1G and 1H are examples where participants under- or over-estimate both angle and distance. Maybe refer to Figs 2A and 2B instead.

*We thank the reviewer for pointing this out and we have changed the text to	refer to Figs2A and 2B.*

(16) Line 210: specifically, with Klatzky et al. -> specifically with Klatzky et al.

*We have implemented this suggestion.*

(17) Lines 223-224: This sentence is unnecessary. 0.87+/-0.5 does not seem that close to 1. I would suggest that it be simply "The normalized walked distance was 0.87 +/- 0.5”.

*We have implemented this suggestion.*

(18) Lines 279-280: Parentheses around stats

*We have implemented this suggestion.*

(19) Line 326: Delete “as well”

*We have implemented this suggestion.*

(20) Line 358: leaving angle constant -> leaving the angles constant

*We have implemented this suggestion.*

(21) Lines 384-386: Very confusing sentence structure.

We thank the reviewer for pointing this out, we have removed this	sentence.

(22) Line 401: model -> models

*We have implemented this suggestion.*

(23) Lines 451,452: subject’s -> subjects’

*We have implemented this suggestion.*

(24) Line 510: guess at it -> guess it

*We have implemented this suggestion.*

(25) Line 512-513: Delete phrase in parentheses. I don’t think it is necessary.

*We have implemented this suggestion.*

(26) Line 578: participant’s -> participants’

*We have implemented this suggestion.*

(27) Line 583: subjects -> participants

*We have implemented this suggestion.*

(28) Line 622: Experiment #1 -> Experiment 1

*We have implemented this suggestion.*
